# Association Between Alcohol Consumption and Risk of Bladder Cancer: A Dose-Response Meta-Analysis of Prospective Cohort Studies

**DOI:** 10.3389/fonc.2021.696676

**Published:** 2021-09-15

**Authors:** Yongfeng Lao, Xiaolong Li, Lijuan He, Xin Guan, Rongxin Li, Yanan Wang, Yanyou Li, Yunchang Wang, Xu Li, Shuai Liu, Zhilong Dong

**Affiliations:** ^1^Second Clinical Medical College, Lanzhou University, Lanzhou, China; ^2^Department of Urology, Lanzhou University Second Hospital, Lanzhou, China; ^3^Key Laboratory of Gansu Province for Urological Diseases, Gansu Nephro-Urological Clinical Center, Lanzhou, China; ^4^Institute of Urology, Lanzhou University Second Hospital, Lanzhou, China

**Keywords:** alcohol, bladder cancer, dose-response, systematic review, meta-analysis

## Abstract

**Background:**

Controversial results of the association between alcohol consumption and risk of bladder cancer were reported by the previous meta-analyses.

**Objective:**

To quantitatively investigate the association between alcohol consumption and risk of bladder cancer based on prospective cohort studies, and explore whether there is potential dose-response relation.

**Method:**

PubMed, EMBASE, the Cochrane Library databases, China Biology Medicine disc (CBM), and Chinese National Knowledge Infrastructure (CNKI) were searched for relevant studies. Categorical meta-analysis was performed for risk estimates of any alcohol consumers versus non-drinkers as well as different drinking degrees (light, moderate, and heavy) versus none. And two-stage generalized least-squares regression and restricted cubic spline, as well as fixed-effects dose-response models, were used for linear and nonlinear dose-response relation exploration.

**Results:**

9 prospective cohort studies including 1,971,396 individuals were finally included. We did not observe a significant association between alcohol intake and the risk of bladder cancer in the entire population. Linear association was detected in those who consumed alcohol from liquor or spirits (P _linear_=0.02). One drink increment each day of alcohol could elevate the risk of bladder cancer by 9% (RR=1.09; 95%CI: 1.01-1.17). Alcohol was a risk factor of bladder cancer for male drinkers (RR=1.23; 95%CI: 1.13-1.35; I^2^=3.7%), while none linear or nonlinear relation was found.

**Conclusion:**

No significant association between alcohol consumption and bladder cancer risk was found in the entire population, but there was a linear dose-response relation in those who consume alcohol from liquor or spirits. Alcohol may elevate the risk of bladder cancer in males in a dose-independent way.

**Systematic Review Registration:**

https://www.crd.york.ac.uk/prospero/, PROSPERO (CRD42020216195).

## Introduction

Bladder cancer is the 10th most commonly diagnosed cancer worldwide, with an estimated 549,000 new cases and 200,000 deaths each year (1). Men are more commonly affected by bladder cancer than women, with incidence and mortality rates of 9.6 and 3.2 per 100,000 in men respectively: about four times those of women globally (1, 2). As bladder cancer carries a large societal burden, identifying its risk factors provides important insight for controlling the high incidence and mortality rates. Cigarette smoking is the most exposure contributing to half the risk of developing the disease as estimated (3, 4). And some environmental exposures, such as aromatic amines and industrial chemicals, have been also linked to bladder cancer in the past decades (5, 6).

As a globally consumed beverage, alcohol consumption has been proved to be associated with many cancers (7). However, the relationship between alcohol consumption and bladder cancer remains perplexing. A meta-analysis of ten cohort studies in Japan showed no evidence of an association between alcohol drinking and bladder cancer risk among men and women (8). But another meta-analysis of case-control and cohort studies suggested that heavy alcohol consumption increased significantly the risk of bladder cancer in men and the Japanese population without significant statistical heterogeneity (9). In a recent large cohort study, the researchers found an association between high intakes of alcohol and the increase in urothelial cell carcinoma (UCC) risk observed in men and smokers that were interpreted as potentially residual confounding by smoking (10). Furthermore, some studies tried to find other reasons to explain the puzzling relation between alcohol consumption and bladder cancer risk. Masaoka et al. found that moderate alcohol drinking among men with flushing was associated with an increased risk of bladder cancer which might support the hypothesis that acetaldehyde derived from alcohol consumption plays an important role in the development of bladder cancer (11). And a case-control study suggested that those with inactive ALDH2 alleles showed an elevated risk of bladder cancer among alcohol drinkers (12).

Though studies sprang up in the past decades, the problem was far from resolved. To our knowledge, there is no systematic review without region restriction of current prospective studies to explore the association between alcohol consumption and risk of bladder cancer, especially whether a dose-response relation exists. Thus, we conducted this dose-response meta-analysis to comprehensively synthesize available prospective studies for exploring the potential dose-response association in the entire and specific populations.

## Method

### Protocol and Registration

We have prospectively registered the protocol of this dose-response meta-analysis on PROSPERO platform (www.crd.york.ac.uk/prospero/) and the registration number is CRD42020216195 (link to details: https://www.crd.york.ac.uk/prospero/display_record.php?RecordID=216195). The Preferred Reporting Items for Systematic Review and Meta-Analysis (PRISMA) was referenced throughout the process of this meta-analysis (13).

### Eligible Criteria

This research intended to assess the association between alcohol consumption and risk of bladder cancer comprehensively based on meta-analysis of prospective studies. PICOS (population, intervention, comparison, outcome, and study design) guideline was strictly followed in this study. Only studies that met the following criteria simultaneously were enrolled in the final systematic review and meta-analysis: (1) Population: the study included participants who were free of bladder cancer and were followed up to investigate the association between alcohol consumption and risk of bladder cancer. And there was no restriction on the comorbidity of participants at the baseline in the original study; (2) Intervention/Comparison: the study included attainable information of different alcohol exposure (Any versus none; or multiple levels of alcohol exposure) of the population. And none restriction was set on the alcohol unit (frequency or quantity); (3) Outcome: alcohol exposure level-specific risk for bladder cancer which was measure by hazard ratio (HR) or relative risk (RR) associated with 95% confidence interval (CI) were reported in the study; (4) Study design: was a prospective cohort study. Additionally, if multiple studies were published based on the same cohort, we then chose that with a larger sample size or longer follow-up time. Studies were excluded if the full-text could not be obtained after trying many approaches such as contacting the corresponding author.

### Search Strategy

To assess the relationship between alcohol consumption and the risk of bladder cancer systematically, we retrieved the published literature to obtain available data as much as possible. The following electronic databases were searched from inception to October 27, 2020: PubMed, EMBASE, Cochrane Library, China Biology Medicine disc (CBM), and Chinese National Knowledge Infrastructure (CNKI). And the following key terms were applied: alcohol, ethanol, beer, wine, liquor, spirit, bladder cancer, etc. (full search strategy was available in **Supplementary Text 1**). Formulation of the retrieve strategy was logically intersected by the keywords of intervention (I) and outcome (O) based on the PICOS guideline to cover comprehensive studies on the association between alcohol consumption and bladder cancer risk. No restrictions on language and publication time were imposed at the retrieval stage.

### Study Selection

Two reviewers independently reviewed all the titles and abstracts of retrieved articles firstly for preliminary inclusion based on pre-set eligible criteria. And then full-text of the literature left at the first stage were checked for the final inclusion by two reviewers independently too. Any disputes arising in the pairing process were resolved by consensus.

### Data Extraction and Quality Evaluation

Two reviewers firstly extracted literature information into a standardized form independently. For each study, the following information was extracted: (1) basic characteristics: first author, publication year, study design, study area, sample size, duration of follow-up, and lost to follow-up rate, etc.; (2) participants details: age, gender, comorbidity, type of bladder cancer, and diagnostic criteria, etc.; (3) details of alcohol exposure: method of alcohol exposure measurement, alcohol categories and unit, etc.; (4) outcomes of each alcohol exposure level: number of events, adjusted confounders, effect size (RR, or HR associated with 95%CI). The extracted data were cross-checked by the two reviewers finally. The Newcastle-Ottawa Scale (NOS) tool (14) was used for risk of bias assessment of cohort studies by two co-authors independently firstly and then cross-checked. The NOS tool contains 8 items which can be categorized into 3 dimensions for cohort studies: selection (4 items, 1 star each), comparability (1 item, up to 2 stars), and outcome (3 items, 1 star each). Research with scores of “0-3”, “4-6” and “7-9” was regarded as “low”, “medium” and “high” quality, respectively (15). Any disputes arising through the pairing process were also resolved by consensus.

### Data Analysis

We conducted categorical meta-analysis firstly for risk estimates of any alcohol consumption versus none. Risk estimates reflective of the same category were combined using the fixed-effect model in the same study and the random-effect model in different studies. Additionally, alcohol exposure was artificially defined qualitatively as light (<12 grams/day), moderate (12-24 grams/day), and heavy (>24 grams/day) based on previous studies (16–18). The pooled results for qualitative categories were compared to observe the variation trend of bladder cancer risk based on alcohol exposure. Then, to conduct dose-response analysis, we assigned the median or mean alcohol intake for alcohol category to each corresponding HR/RR. When the median or mean alcohol intake was not reported, the midpoints of the upper and lower boundaries in each category were defined as the median intake. When the lowest or the upper boundary was open-ended, we set the lower boundary to zero and assumed the upper boundary had the same amplitude as the adjacent category (16, 19, 20). Since alcohol intake was not measured by unified units in various included studies, heterogeneous units were converted to grams/day for analysis finally. For studies using “drink” as the alcohol unit, we assumed that one drink contains 12 g pure alcohol the same as previous studies if included studies did not report specific conversion criteria (16, 20). For those studies exploring specific alcoholic beverages such as beer, wine, or spirits, we calculated the amount of pure alcohol contained in a specific drink based on the percentage of alcohol volume (% vol) if it was reported. And we assumed about 4% vol were contained in beer, 12% vol in wine, and 40% in spirits based on the previous study if alcohol volume were not reported (21), though the ethanol content in different alcoholic drinks could be varied from country to country.

The methods named two-stage generalized least-squares regression (GLST) and restricted cubic spline as well as fixed-effects dose-response models were used for linear and nonlinear dose-response meta-analysis (22, 23). As the reference category is supposed to be the least exposure level to fit for later dose-response analysis, we regarded the lowest category as the reference and recalculate the effect size using the method by Hamling et al. for studies wherein the reference group was not the lowest category (24). Sensitive analysis was performed by omitting one study each time and excluding studies that using the assumed standard to obtain the pure alcohol amount for different alcoholic types. Subgroup analysis was performed based on gender, alcohol source, smoking status, and different regions for more specific results or to find potential heterogeneity. Egger test was used to assess publication bias if included studies were more than ten (25, 26). The I^2^ statistic was used to measure the heterogeneity among included studies. I^2^>50% and P<0.05 were defined as significant heterogeneity. All statistical analyses were performed in Stata version 15.0 (Stata Corp, College Station, TX, USA), with two tail-tailed P<0.05 for statistical significance.

## Results

### Literature Search

**Figure 1:**
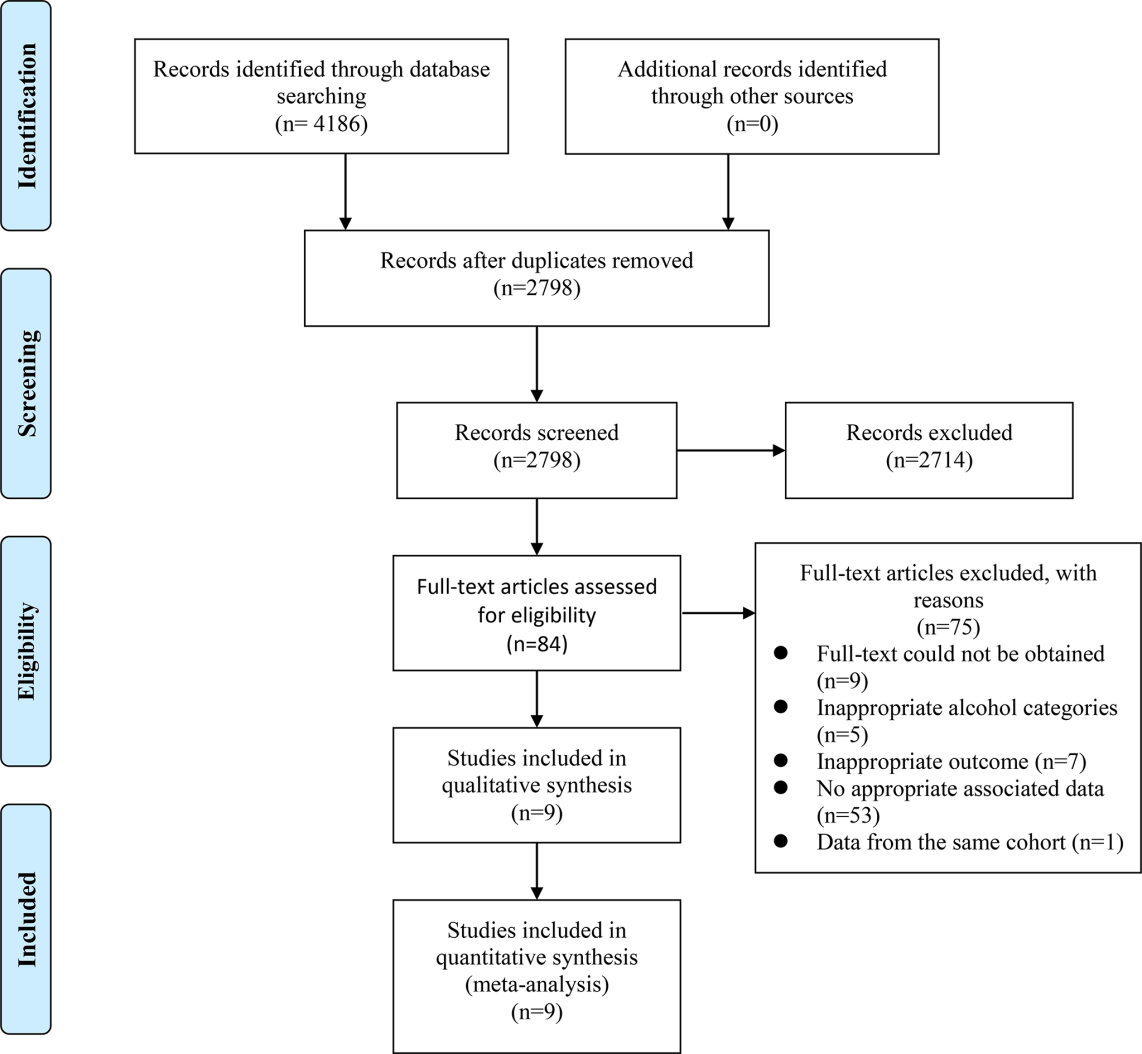
Flow diagram of the literature search and study selection process.

The selection process was presented in a flow diagram (**Figure 1**). We identified 4,186 articles during the initial electronic database search, of which 2,798 records were left after removing duplication. 2,714 records were excluded after reviewing titles and abstracts, leaving 84 papers for the full-text check. Finally, 75 studies were excluded and a total of 9 prospective cohort studies were enrolled (10, 11, 27–33). Titles of excluded articles after full-text check were provided in **Supplementary Table 1**.

### Study Characteristics

All the eligible 9 studies were prospective cohort design and published in English. In total, 1,971,396 individuals with 4,385 bladder cancer cases were included in these cohorts. 5 cohorts were conducted in the USA, 3 cohorts were conducted in Europe, and 1 cohort was conducted in Japan. 2 studies (28, 29) included males only while another 2 studies (32, 33) just considered females. All studies were considered high quality (NOS scores≧7). The basic characteristics of included studies were provided in **Table 1**. Detailed scores for each item of NOS of each study can be found in **Supplementary Table 2**.

**Table 1:** Basic characteristic of studies included in the systematic review and dose-response meta-analysis (M: male; F: female).

Study	Study region	Sample size	Cases	Age at baseline (year)	Sex (male)	Follow-up time (year)	Alcohol categories	Adjusted confounders	NOS scores
Mills (27)	USA	34198	52	25~100	NA	6 (max)	Never Any	Age, sex, and smoking	9
Chyou (28)	USA	7995	96	46~68	100%	22 (max)	0 <15 grams/day ≧15 grams/day	Age, smoking	8
Michaud (29)	USA	47909	252	40~75	100%	10 (max)	<1 glass/month 1-3 glass/month 1-4 glass/week 5 glass/week-1 glass/day ≧2 glass/day	Age, geographic region, pack-years of smoking, current smoking status, energy intake, and intake of fruits and vegetables	8
Zeegers (30)	Netherlands	3170	594	55~69	50.19%	6.3 (max)	None <5 grams/day 5-15 grams/day 15-30 grams/day ≧30 grams/day	Age, smoking (smoking status, amount, and duration)	8
Djoussé (31)	USA	10125	126	40.3 ± 10.4/5~70	47.23%	27.3±10.1	0 0.1–6.0 grams/day 6.1–12.0 grams/day 12.1–24.0 grams/day 24.1–48.0 grams/day ≧48.0 grams/day	Age, sex, cohort, smoking status, pack-years of cigarette smoking	9
Allen (32)	UK	1280296	928	55 (mean)	0	7.2 (mean)	Nondrinkers ≦2 drinks/week 3-6 drinks/week 7-14 drinks/week ≧15 drinks/week	Age, region of residence, socioeconomic status, body mass index, smoking, physical activity, use of oral contraceptives, and hormone replacement therapy	9
Botteri (10)	Europe	476160	1802	51.2 (mean)	29.90%	13.9 (mean)	Nondrinker 0–6(M)/0–3(F) grams/day 6–12(M)/3–12(F) grams/day 12–24 grams/day 24–60 grams/day 60–96(M)/60(F) grams/day 96(M) grams/day	Age, sex, smoking status, energy intake, body mass index, physical activity and educational level completed	9
Masaoka (11)	Japan	95915	464	52.2 ± 8.0/40~69	47.59%	18.2 (mean)	Non/Occasional drinker 1–150 grams/week 151–300 grams/week 301–450 grams/week >450 grams/week	Age, sex, area, smoking	9
Park (33)	USA	15628	71	51.8 ± 6.8	0	NA	0 0.1–4.9 grams/day 5.0–14.9 grams/day 15.0–29.9 grams/day ≧30.0 grams/day	Age, smoking status, BMI, physical activity, menopause status, postmenopausal hormone use, history of hypercholesterolemia, hypertension or type 2 diabetes	7

### Categorical Meta-Analysis

**Figure 2:**
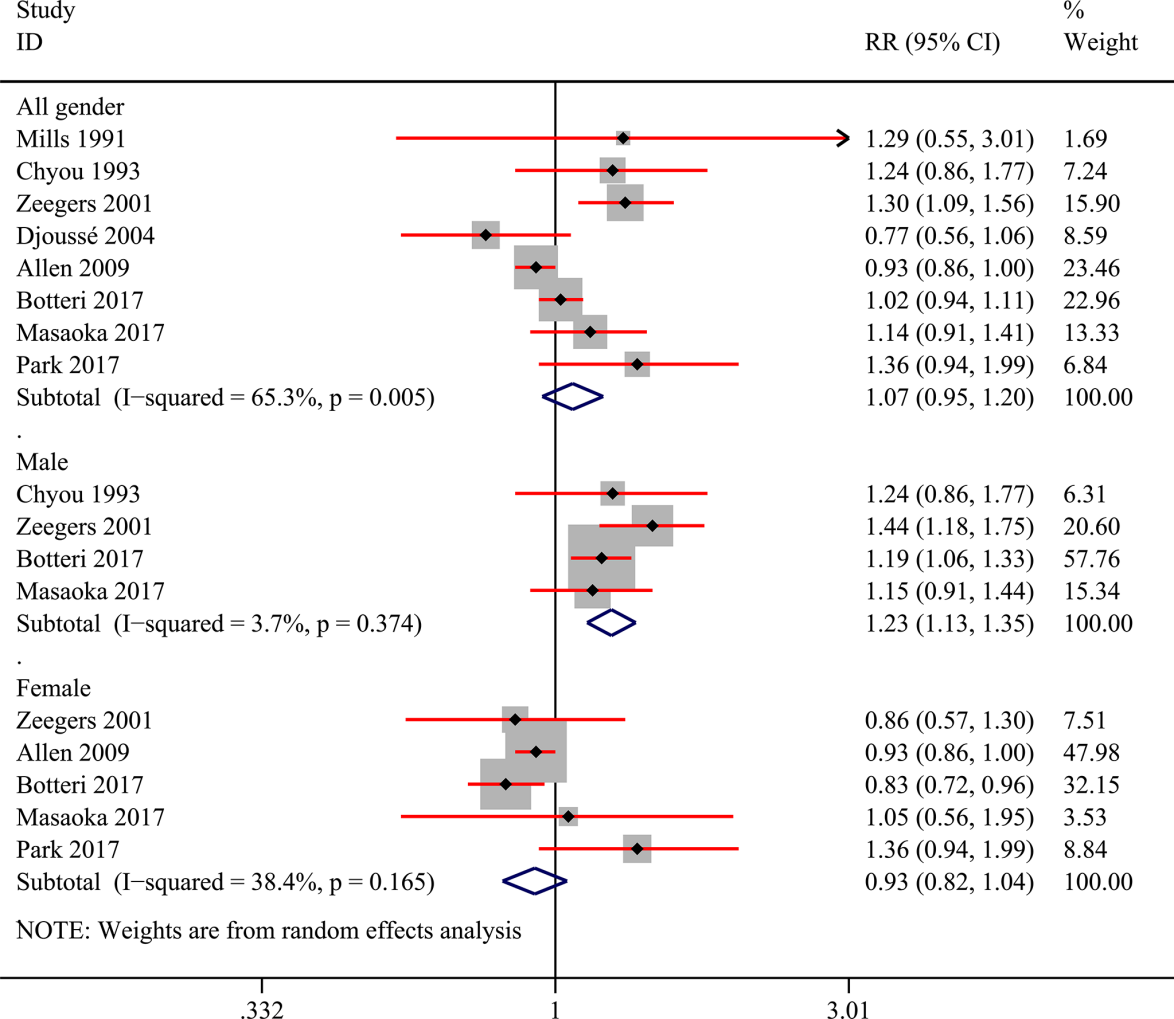
Forest plot of relative risk (RR) of bladder cancer for alcohol consumption (Any *versus* none) in the entire population and different gender subgroups.

**Figure 3:**
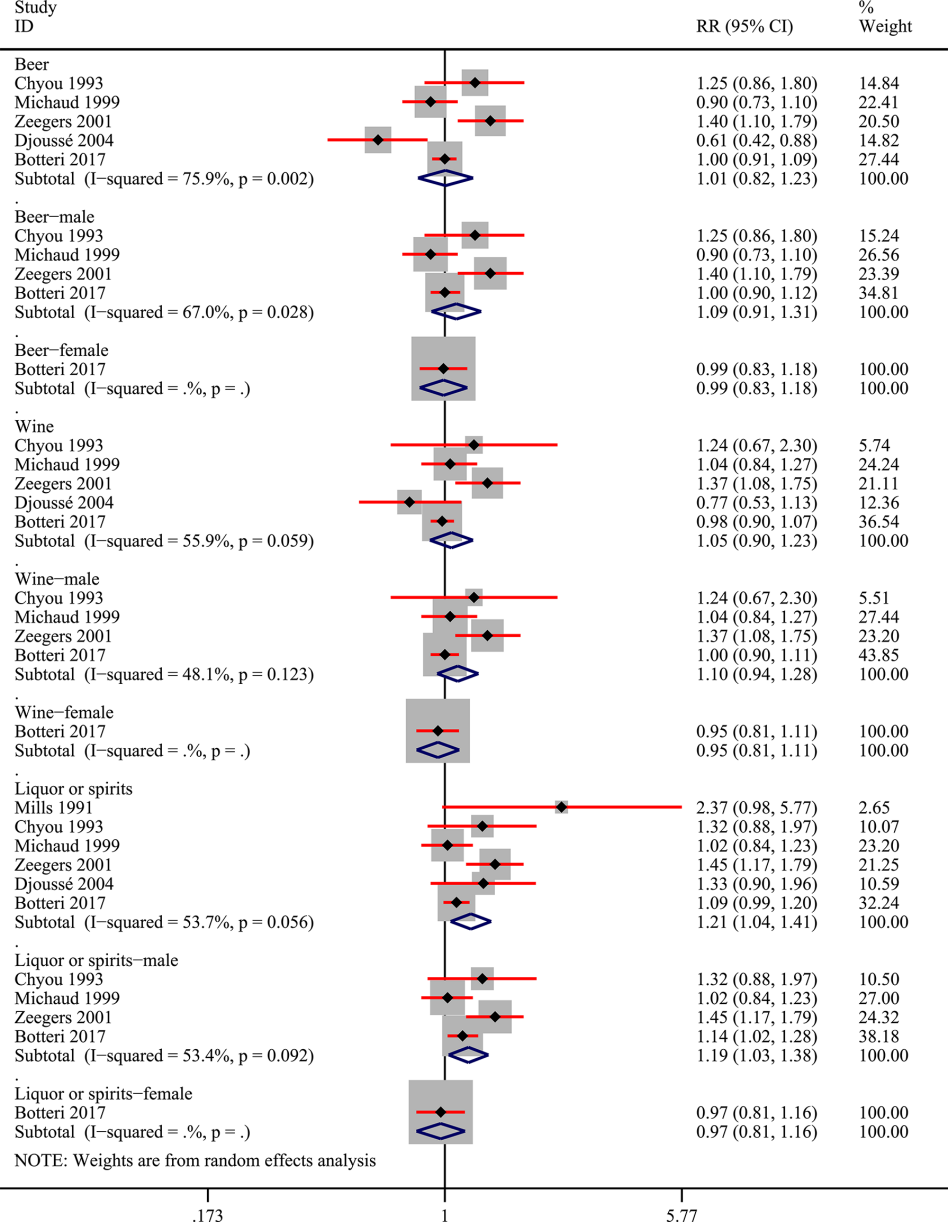
Forest plot of relative risk (RR) of bladder cancer for alcohol consumption (Any *versus* none) of different sources in the entire population and different gender subgroups.

Among the 9 included studies, two studies reported the risk data of alcohol consumers compared with non-drinkers directly (11, 27). Data of multiple levels of alcohol exposure in another 7 studies were then converted into dichotomous levels (any versus none) indirectly to explore the risk differences between alcohol consumers and non-drinkers. Firstly, 8 studies (10, 11, 27, 28, 30–33) were included to pool the risk estimates of any alcohol intake versus none. And the results showed no significantly changed risk of bladder cancer in alcohol consumers, but the heterogeneity was significant (RR=1.07; 95%CI: 0.95-1.20; I^2^=65.3%) (**Figure 2**). Then, we conducted subgroup analysis based on gender, and the results showed that alcohol consumption could elevate the risk of bladder cancer in the male population (RR=1.23; 95%CI: 1.13-1.35; I^2^=3.7%), which was not observed in women (RR=0.93; 95%CI: 0.82-1.04; I^2^=38.4%) (**Figure 2**). Heterogeneity in these two subgroups presented a significant reduction. Furthermore, we explored potential risk differences of bladder cancer when consumption of alcohol came from different types of alcoholic drinks (beer, wine, liquor or spirits), taking into account the influence of gender. The results indicated that alcohol from liquor or spirits in the entire population (RR=1.21; 95%CI: 1.04-1.41; I^2^=53.7%) and male (RR:1.19; 95%CI: 1.03-1.38; I^2^=53.4%) could elevate the risk of bladder cancer (**Figure 3**). Heterogeneity in the subgroups of beer and wine was statistically significant while it was near the critical value in the liquor or spirits subgroup and it was reduced to a certain degree when the male subgroup was considered separately. Only one study (10) reported the data of bladder cancer risk when consumption of alcohol was from different types of alcoholic drinks in females which were also presented in **Figure 3**. And it suggested that alcohol consumption from different types of alcoholic drinks seemed not to be significantly related to the risk of bladder cancer in females. Subgroup analysis of different regions (Europe or the USA) and smoking status was also performed and no statistical difference was found between Europe and the USA, or never smokers and past or current smokers (**Supplementary Figures 1** and **2**).

**Figure 4:**
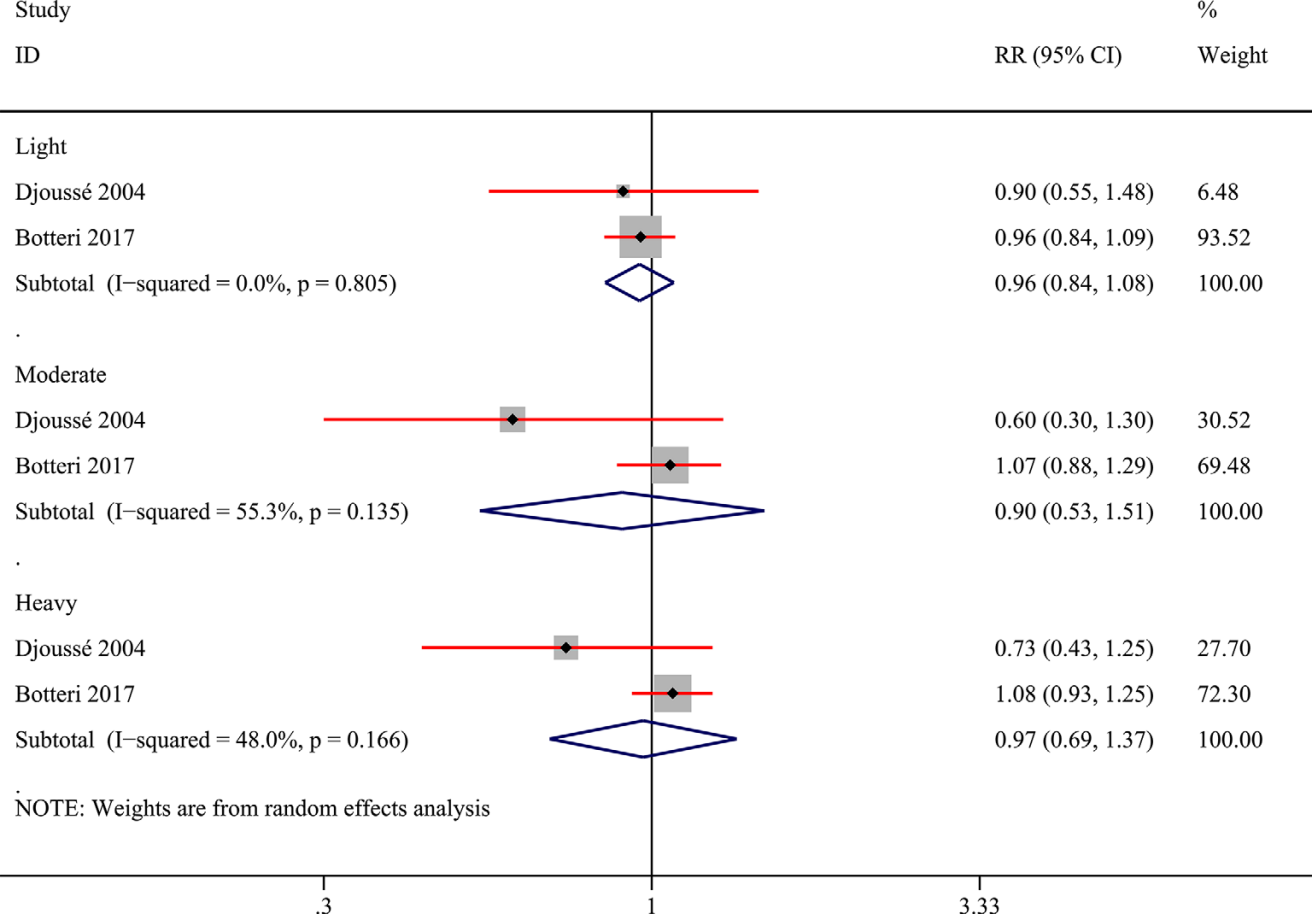
Forest plot of relative risk (RR) of bladder cancer for alcohol consumption (Light, moderate, and heavy *versus* none) in the entire population.

Additionally, we tried to convert various alcohol exposure in each study into three patterns (light, moderate, and heavy) based on pre-set criteria. When converting the units of each study into “grams/day”, two studies (28, 29) utilized the assumed concentration standard (21) to calculated alcohol amounts from different alcoholics. Two studies (10, 31) could be properly classified in the last and the pooled results showed no statistical difference (**Figure 4**). And we presented the risk data of different levels of alcohol consumption in various subgroups in **Supplementary Table 3** though the available data could be only obtained from one study (10). Heavy drinking was risky for bladder cancer in males (RR:1.23; 95%CI: 1.02-1.48) while light drinking was a protective factor for bladder cancer in females (RR:0.79; 95%CI: 0.66-0.96) (**Supplementary Table 3**). No other statistically significant results were found in the other subgroups.

### Dose-Response Meta-Analysis

**Figure 5:**
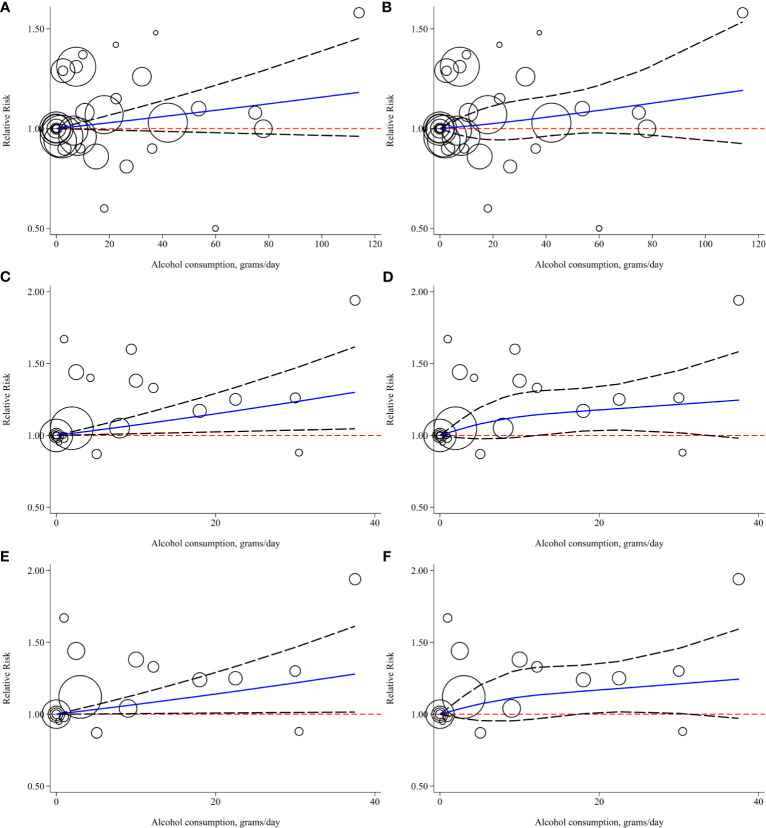
Linear and nonlinear fitting of alcohol consumption and risk of bladder cancer **(A)** linear association in entire population; **(B)** nonlinear association in entire population; **(C)** linear association in entire population who consumed alcohol for liquor or spirits; **(D)** nonlinear association in entire population who consumed alcohol from liquor or spirits; **(E)** linear association in males who consumed alcohol form liquor or spirits; **(F)** nonlinear association in males who consumed alcohol from liquor or spirits. (Bubbles were weighted by the number of cases at each dose point).

To conduct the dose-response meta-analysis, alcohol units in two studies were converted from “drink” to “gram” based on the criteria self-reported in each study respectively while the pure alcohol amount from different alcoholics in two studies (28, 29) was calculated based on pre-set concentration standard (21) for further subgroup analysis. Firstly, 7 studies were pooled for dose-response relationship exploration without alcohol type and gender restrictions (10, 11, 28, 30–33). However, there was no evidence of a linear (P _linear_=0.11, **Figure 5A**) or nonlinear (P _nonlinear_=0.28, **Figure 5B**) association between alcohol consumption and risk of bladder cancer. Then, we conducted subgroup analysis based on gender, alcohol source in the entire population and different genders, smoking status, and region. All the linear and nonlinear fitting results were presented in **Table 2**. We also calculated the relative risk of each 12 g (1 drink) increment of alcohol in each subgroup (**Table 2**). Alcohol consumption from liquor or spirits in the entire population showed a mild linear association between alcohol consumption and risk of bladder cancer (P _linear_=0.02, **Figure 5C**) while no nonlinear association was observed (**Figure 5D**). One drink increment of alcohol consumption could elevate bladder cancer risk by 9% (RR=1.09; 95%CI: 1.01-1.17). Similarly, in the male subgroup of alcohol consumption from liquor or spirits, mild linear association (P _linear_=0.04, **Figure 5E**) and no nonlinear association were observed (**Figure 5F**). Bladder cancer risk was elevated by 8% for one drink increment (RR=1.08; 95%CI: 1.00-1.17). Only one study reported the risk data of bladder cancer with different alcohol sources in females as well as of past or current smokers (10). Linear and nonlinear fitting was also performed for the data from such a single study, and the results were presented in **Table 2**. A mild linear association was found in past or current smokers while no linear or nonlinear relationship was observed in females with different alcohol sources (**Table 2**).

**Table 2:** Summary of subgroup analysis results based on fixed-effect linear and nonlinear (3 knots) dose-response meta-analysis. Bold values mean results with statistical significance.

Subgroup	Number of cohorts	Linear model				Nonlinear model		
		RR	95%CI	P heterogeneity	P model	P heterogeneity	P model	P knots
**Gender**								
Male	4	1.02	1.00-1.05	0.97	0.09	0.27	0.14	0.31
Female	5	0.98	0.92-1.04	0.36	0.48	0.82	0.36	0.21
**Alcohol source**								
Beer	5	1.03	0.98-1.08	0.29	0.25	0.66	0.25	0.22
Male	4	1.03	0.98-1.08	0.69	0.27	0.76	0.44	0.51
Female	1	1.11	0.92-1.35	–	0.29	0.37	0.26	0.21
Wine	4	1.01	0.97-1.05	0.79	0.71	0.42	0.61	0.36
Male	3	1.01	0.96-1.05	0.94	0.78	0.22	0.79	0.53
Female	1	1.01	0.91-1.12	–	0.84	0.69	0.95	0.78
Liquor or spirits	5	**1.09**	**1.01-1.17**	0.12	**0.02**	0.32	0.04	0.40
Male	4	**1.08**	**1.00-1.17**	0.19	**0.04**	0.20	0.09	0.52
Female	1	1.04	0.83-1.31	–	0.73	0.59	0.94	1.00
**Region**								
USA	3	0.97	0.87-1.07	0.37	0.51	0.79	0.49	0.32
Europe	3	1.02	0.99-1.04	0.01	0.15	0.08	0.35	0.88
**Smoking status**								
Never smokers	2	1.01	0.92-1.10	0.10	0.88	0.19	0.57	0.30
Past or current smokers	1	**1.03**	**1.00- 1.06**	–	**0.03**	0.49	0.08	0.69

### Sensitivity Analysis

**Figure 6:**
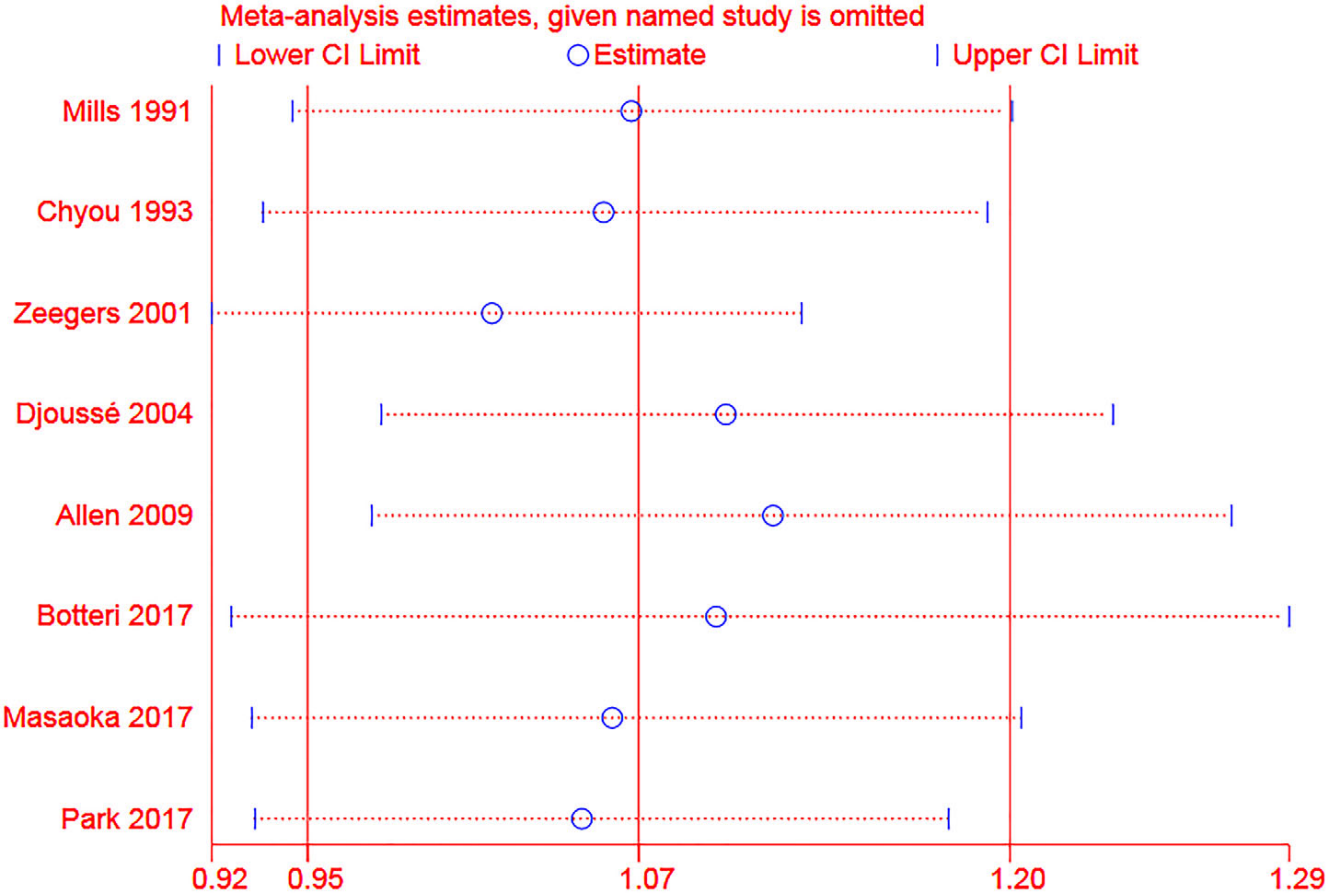
Sensitivity analysis of the association between alcohol consumption (any *vs* none) and risk of bladder cancer (The two ends of the dotted lines represented the 95%CI).

For the sensitivity analysis of categorical meta-analysis, the total result was not significantly changed when one study was omitted each time (**Figure 6**). For dose-response meta-analysis, two studies (28, 29) were excluded for sensitivity analysis because alcohol amounts from specific alcoholic drinks were calculated based on the pre-set concentration standard in these studies (21). All the results were not significantly changed. And we presented the results of the linear and nonlinear fitting curve of alcohol consumption from liquor or spirits and bladder cancer risk of the entire and male population in **Supplementary Figure 3**. As there were less than ten studies included in this meta-analysis, publication bias and meta-regression were not explored (25, 26).

## Discussion

In this dose-response meta-analysis of prospective cohorts, we systematically evaluated the dose-response relation between alcohol consumption and risk of bladder cancer. Although alcohol consumption seemed to have no association with bladder cancer in the entire population, elevated risk was found in specific population that was male and individuals whose alcohol source was from liquor or spirits. Furthermore, a mild linear association was observed firstly in those who consumed alcohol from liquor or spirits.

The association between modifiable lifestyles and the risk of neoplastic and chronic diseases of the genitourinary system has attracted researchers’ attention for a long time (34–37). And lifestyle interventions were found to improve clinical outcomes for cancer or chronic medical conditions, either directly or indirectly (34, 38–40). The bidirectional relationship between lifestyle factors and disease might be systemic and multifaceted, for which lifestyle interventions might have a positive impact on overall health in addition to optimizing the disease-specific outcomes (41, 42). As a common additive to beverages, the relation of alcohol and bladder cancer risk was still contradictory though much cancer was linked with alcohol consumption (7, 43). Past reviews of epidemiological data on alcohol consumption and bladder cancer risk concluded no association and attributed the moderate increase in risk observed in some investigations to residual confounding by smoking, or to an association between alcohol, coffee, and yet unidentified risk factors for bladder cancer (44, 45). Several meta-analyses were also conducted to explore the relationship. Pelucchi et al. carried on a meta-analysis mainly including case-control studies which didn’t find any material association between alcohol consumption and bladder cancer risk using data that adjusted tobacco smoking (46). But another meta-analysis of case-control and cohort studies found both beer and wine consumption exhibited a negative dose-response relationship with bladder cancer risk though the heterogeneity was significant (47). In the contrast, Hong et al. didn’t find any significant association between all three beverage types (beer, wine, liquor) and the risk of bladder cancer (48). In this dose-response meta-analysis that only enrolled prospective cohorts studies, we found that alcohol drinking for liquor or spirits in the entire population and males could elevate the risk of bladder cancer which was consistent with Vartolomei and his colleagues’ study (9). Furthermore, since tobacco smoking was adjusted in all the studies, and to make our results less influenced by residual confounding by smoking, we performed subgroup analysis in smokers and past or current smokers. No significant difference was found in the two subgroups after data synthesis. But until now, data of past or current smokers was still scarce for dose-response meta-analysis, fitting results from a single study (10) suggested a weak linear correlation, indicating more data is needed in the future to control residual confounding of smoking more rigorously.

In the categorical meta-analysis, we found that male alcohol consumers had a higher risk of bladder cancer than non-drinkers which was against Hong and his colleagues’ research (48). When alcohol exposure was converted into various degrees (light, moderate, and heavy), the meta-analysis showed no significant impact of alcohol intake on the bladder cancer risk in the entire population. Similarly, Vartolomei and his colleagues found an increased risk of bladder cancer in heavy male alcohol consumers which could not be observed in the entire population (9). Meta-analysis was infeasible because only one study (10) contained the data of different subgroups. The single research suggested that heavy drinking elevated bladder cancer risk of males, supporting the meta-analysis results without classification of alcohol consumption. It also indicated that light female drinkers might have a lower risk of bladder cancer. But the perception of the relationship between alcohol consumption and bladder cancer risk in females may not change because the study was also the only one with statistically significant results included in the categorical meta-analysis comparing alcohol consumers versus non-drinkers in females (**Figure 2**), and no significant relationship in females was found in the dose-response exploration too. Actually, the qualitative classification criteria of alcohol consumption were not always consistent (18, 49). Alcohol exposure in the original studies also varied with sample size and measurement (50). It might be a hindrance to exploring the relationship between alcohol consumption and disease risk. Over the past several years, dose-response meta-analyses provided a reliable way to utilize these multitudinous epidemiological data (51). However, though findings were not always consistent, previous negative results from large sample studies might be suggestive, and previous narrative overviews tended to interpret the positive results as a differential confounding effect of tobacco smoking–the major risk factor for bladder cancer (27, 29, 44, 45). Studies in the field might gradually dwindle as a result, explaining an important proportion of articles that came from 15 years ago were enrolled when we conducted this dose-response meta-analysis.

Our dose-response relation fitting did not show any significant relation in males except in the population who consumed alcohol from liquor or spirits. But the statistical significance of the linear dose-response model was closed to the critical value which indicated that the results should be interpreted cautiously. Gender differences in alcohol-related cancer risk have been observed for a long time (52, 53). Some researchers attributed the carcinogenicity of alcohol to its metabolite-acetaldehyde (ACE) accumulation which was associated with flushing response (11, 54). However, a meta-analysis by Zhang et al. suggested that facial flushing response was associated with cancer risk in men, yet not existed among women (55) which suggested a complex mechanism in gender difference of alcohol-induced cancer risk. All in all, though the same as found in many studies–female bladder cancer risk seemed not to be influenced by alcohol consumption, risk of bladder cancer could be elevated in male drinkers without linear or nonlinear increasing trend. It may explain, in part, men are more likely to develop bladder cancer than women (1, 2, 56). The results might be due to complex metabolic processes of alcohol in the body and further molecular epidemiology should be conducted to validate and interpret such phenomenon.

When exploring the heterogeneity source of the total results of categorical meta-analysis, we found that heterogeneity had been significantly reduced in the USA subgroup but elevated in the Europe subgroup. It suggested that the association between alcohol consumption and bladder cancer risk might be different in some regions. Hong et al. reported a protective effect of alcohol consumption for bladder cancer in America and inverse associations in Europe and Asia (48). However, our meta-analysis of prospective cohort studies found no relationship between alcohol consumption and risk of bladder cancer in the USA and Europe while results of Europe showed significant heterogeneity. Additionally, Vartolomei and his colleagues revealed that heavy alcohol consumption increased significantly the risk of bladder cancer in the Japanese population (9) which conflicted with a meta-analysis of cohort studies conducted in Japan that null relationship was observed in both males and females (8). In this dose-response meta-analysis, we could not identify several eligible cohorts included in the above meta-analysis based on the electronic database retrieval because some cohort results were published in Japanese. Thus, we could not exam the association between alcohol consumption and bladder cancer risk in Japanese reported by Vartolomei et al. (9) and Masaoka et al. (8) and explore the dose-response relationship due to data limitation. Anyway, our results did not reveal any active association in specific regions, however, results in Europe and Japan should be validated and fleshed out in the further.

Direct contact with carcinogens excreted in the urine was regarded as a potential cause of bladder cancer which might be related to the fact that most cases of bladder cancer occur in the cells of the bladder innermost lining (29, 57). Such exposure may be reduced by high consumption of fluids that can dilute the urine and reduce contact time through increased frequency of urination (29). However, the hypothesis was hard to verify since results considering total fluid intake were inconsistent (48, 58, 59). Furthermore, when considering the specific type of fluid, such as alcohol, there might be not a just simple mechanical effect that dilutes the urine and reduces contact time. A complex interaction is predicted to exist between alcohol as well as its metabolite byproduct and bladder tissue and each layer of bladder cells besides direct contact, thus, toxicity is not negligible. The primary breakdown product of ethanol in the body, acetaldehyde, was shown to bind to proteins and alter their structures and functions–particularly for enzymes involved in DNA repair and glutathione metabolism, thereby contributing to cancer risk (60). Along this line of thought, key enzymes in ethanol metabolism caught researchers’ attention. For bladder cancer, a matched case-control study concluded that those with the ALDH2 Glu/Lys and ADH1B Arg+ genotypes were at increased risk of bladder cancer, but prospective studies are needed to validate such a conclusion (12). Non-invasive, sensitive, fast, and inexpensive hematological biomarkers are urgently demanding for early diagnosis and personalized medicine of bladder cancer (61). And liquid biopsy of circulatory markers such as systemic inflammatory markers and circulating tumor cells showed great potential for early diagnosis of bladder cancer and prediction of oncologic outcomes (62–65). Though we were in the research framework that considered a single lifestyle factor and bladder cancer risk, polymorphisms of key ethanol metabolism genes, as well as their interactions with the body liquid environment, might be the crux for some time to seek molecular markers besides clinical parameters for predicting alcohol-induced cancer risk as well as oncologic outcomes. Furthermore, some research found specific compositions in different alcoholic drinks, such as phenolic compounds, namely Xanthohumol in beer and Resveratrol in wine, had a potential protective effect for cancer (66, 67). We had recognized a combined action between ethanol and certain composition in various alcoholic drinks, thus, to avoid conceptual confusion, we calculated the pure alcohol amount from different alcoholic drinks, rather than used the amount of each type of drink as “alcohol consumption” in the dose-response analysis. In other words, alcohol-induced cancer risk may be a clinical relation in the interaction of multiple components.

The primary strength of this study was its dose-response analysis of prospective cohort studies which could better assess the strength of causal relation (68). Another significant strength was its total large sample size of included high-quality studies. There were also several limitations to this study. Firstly, due to data limitation, we could not perform subgroup meta-analysis based on different types of bladder cancer and different alcohol sources in females, as well as exploring the dose-response relationship in past or current smokers. Secondly, we could not provide synthetical results of any subgroups when alcohol consumption was defined as three categories (light, moderate, and heavy). Thirdly, we could not exclude the potentially spurious association caused by some confounders as adjusted confounders were inconsistent in different studies.

## Conclusion

No significant association between alcohol consumption and bladder cancer risk was found in the entire population, but there was a linear dose-response relation in those who consume alcohol from liquor or spirits. Alcohol may elevate the risk of bladder cancer in males in a dose-independent way. Further high-quality studies should be conducted to validate our results and further explore other specific population groups and determine potential regulator genes based on molecular epidemiology.

## Data Availability Statement

The original contributions presented in the study are included in the article/**Supplementary Material**. Further inquiries can be directed to the corresponding author.

## Author Contributions 

YFL and XLL designed the program, searched and reviewed the studies, analyzed the data, and were in charge of the manuscript. LJH, XG, RXL, YNW, YYL, and YCW extracted the data and wrote part of the manuscript. XL and SL revised part of the manuscript after the peer-review. ZLD directed the project, contributed to the discussion, reviewed, and edited the manuscript. All authors contributed to the article and approved the submitted version.

## Conflict of Interest

The authors declare that the research was conducted in the absence of any commercial or financial relationships that could be construed as a potential conflict of interest.

## Publisher’s Note

All claims expressed in this article are solely those of the authors and do not necessarily represent those of their affiliated organizations, or those of the publisher, the editors and the reviewers. Any product that may be evaluated in this article, or claim that may be made by its manufacturer, is not guaranteed or endorsed by the publisher.
