# Supplementary material for: Association Between Alcohol Consumption and Risk of Bladder Cancer: A Dose-Response Meta-Analysis of Prospective Cohort Studies
Source: Front Oncol. 2021 Sep 15;11:696676. doi: 10.3389/fonc.2021.696676 (PMC8479110; doi:10.3389/fonc.2021.696676)
Supplement: Supplementary file 1 [file DataSheet_1.docx]

Association between alcohol consumption and risk of bladder cancer: a dose-response meta-analysis of prospective cohort studies

Yongfeng Lao^1,2,3,4,†^, Xiaolong Li^1,2,3,4,†^, Lijuan He^1^, Xin Guan^1,2,3,4^, Rongxin Li^1,2,3,4^, Yanan Wang^1,2,3,4^, Yanyou Li^1,2,3,4^, Yuchang Wang^1,5^, Zhilong Dong^1,2,3,4,*^

^1^Second Clinical Medical College, Lanzhou University, Lanzhou, 730000, Gansu, People’s Republic of China

^2^Department of Urology, Lanzhou University Second Hospital, Lanzhou, 730000, Gansu, People’s Republic of China

^3^Key Laboratory of Gansu Province for Urological Diseases, Gansu Nephro-Urological Clinical Center, Lanzhou, 730000, Gansu, People’s Republic of China

^4^Institute of Urology, Lanzhou University Second Hospital, Lanzhou 730030, Gansu, People’s Republic of China

^5^Central South University Xiangya Hospital, Changsha, 410012, Hunan, People’s Republic of China

^†^These authors have contributed equally to this work and share the first authorship.

^*^Corresponding author: Zhilong Dong, Department of Urology, Lanzhou University Second Hospital, Lanzhou, 730000, Gansu, People’s Republic of China. Email: dzl1997.student@sina.com.

**CONTENT**

| Title | Page |
| --- | --- |
| **Supplementary Text 1** Searching strategy | 2-3 |
| **Supplementary Table 1** Studies excluded with reasons | 4-7 |
| **Supplementary Table 2** Quality assessment of included studies (Newcastle Ottawa Scale) | 8 |
| **Supplementary Table 3** Summary of the risk of bladder cancer with different alcohol consumption (light, moderate, and heavy) in different subgroups | 9 |
| **Supplementary Figure 1** Forest plot of relative risk (RR) of bladder cancer for alcohol consumption (Any versus none) in different regions | 10 |
| **Supplementary Figure 2** Forest plot of relative risk (RR) of bladder cancer for alcohol consumption (Any versus none) in population with different smoking status | 11 |
| **Supplementary Figure 3** Sensitivity analysis of linear and nonlinear fitting of alcohol consumption and risk of bladder cancer (a: linear association in entire population who consumed alcohol for liquor or spirits; b: nonlinear association in entire population who consumed alcohol from liquor or spirits; c: linear association in males who consumed alcohol form liquor or spirits; d: nonlinear association in males who consumed alcohol from liquor or spirits) (Bubbles were weighted by the number of cases at each dose point) | 12 |

**Supplementary Text 1** Searching strategy

**PubMed**

((((((Alcohol Drinking[MeSH Terms]) OR (Ethanol[MeSH Terms])) OR (Blood Alcohol Content[MeSH Terms])) OR (Binge Drinking[MeSH Terms])) OR (Drinking Behavior[MeSH Terms])) OR ((((((((((((((((ethanol[Title/Abstract]) OR (alcohol*[Title/Abstract])) OR (blood alcohol level[Title/Abstract])) OR (blood alcohol content[Title/Abstract])) OR (wine*[Title/Abstract])) OR (liquor*[Title/Abstract])) OR (spirit*[Title/Abstract])) OR (beer*[Title/Abstract])) OR (beverage*[Title/Abstract])) OR (alcohol drinking[Title/Abstract])) OR (drinking behavior[Title/Abstract])) OR (alcohol consumption[Title/Abstract])) OR (alcohol intake[Title/Abstract])) OR (drink*[Title/Abstract])) OR (drunk*[Title/Abstract])) OR (binge drinking[Title/Abstract]))) AND (((((bladder[Title/Abstract]) OR (urocyst[Title/Abstract])) AND ((((cancer*[Title/Abstract]) OR (neoplas*[Title/Abstract])) OR (tumor*[Title/Abstract])) OR (carcinoma*[Title/Abstract]))) OR (urinary bladder neoplasms[Title/Abstract])) OR (Urinary Bladder Neoplasms[MeSH Terms]))

**EMBASE**

#1 'bladder cancer'/exp OR 'bladder tumor'/exp OR 'bladder carcinoma'/exp

#2 'urinary bladder neoplasms':ab,ti OR 'urinary bladder neoplasm':ab,ti

#3 bladder:ab,ti OR urocyst:ab,ti

#4 cancer*:ab,ti OR neoplas*:ab,ti OR tumor*:ab,ti OR carcinoma*:ab,ti

#5 #3 AND #4

#6 #1 OR #2 OR #5

#7 'drinking behavior'/exp OR 'alcohol'/exp OR 'alcohol blood level'/exp OR 'binge drinking'/exp OR 'alcohol consumption'/exp

#8 ethanol:ab,ti OR alcohol*:ab,ti OR 'blood alcohol level':ab,ti OR 'blood alcohol content':ab,ti OR wine*:ab,ti OR liquor*:ab,ti OR spirit*:ab,ti OR beer*:ab,ti OR beverage*:ab,ti OR 'alcohol drinking':ab,ti OR 'drinking behavior':ab,ti OR 'alcohol consumption':ab,ti OR 'alcohol intake':ab,ti OR drink*:ab,ti OR drunk*:ab,ti OR 'binge drinking':ab,ti

#9 #7 OR #8

#10 #6 AND #9

**Cochrane Library**

#1 MeSH descriptor: [Urinary Bladder Neoplasms] explode all trees

#2 MeSH descriptor: [Alcohol Drinking] explode all trees

#3 MeSH descriptor: [Ethanol] explode all trees

#4 MeSH descriptor: [Blood Alcohol Content] explode all trees

#5 MeSH descriptor: [Binge Drinking] explode all trees

#6 MeSH descriptor: [Drinking Behavior] explode all trees

#7 (urinary bladder neoplasms):ti,ab,kw

#8 (bladder*):ti,ab,kw OR (urocyst):ti,ab,kw

#9 (cancer*):ti,ab,kw OR (carcinoma*):ti,ab,kw OR (neoplas*):ti,ab,kw OR (tumo?r*):ti,ab,kw

#10 #8 AND #9

#11 #1 OR #7 OR #10

#12 (alcohol drinking):ti,ab,kw OR (ethanol):ti,ab,kw OR (blood alcohol content):ti,ab,kw OR (binge drinking):ti,ab,kw OR (drinking behaviour):ti,ab,kw (Word variations have been searched)

#13 (alcohol*):ti,ab,kw OR (blood alcohol level):ti,ab,kw OR (wine*):ti,ab,kw OR (liquor*):ti,ab,kw OR (spirit*):ti,ab,kw (Word variations have been searched)

#14 (beer*):ti,ab,kw OR (beverage*):ti,ab,kw OR (alcohol consumption):ti,ab,kw OR (alcohol intake):ti,ab,kw (Word variations have been searched)

#15 (drunk*):ti,ab,kw OR (drink*):ti,ab,kw (Word variations have been searched)

#16 #2 OR #3 OR #4 OR #5 OR #6 OR #12 OR #13 OR #14 OR #15

#17 #11 AND #16

**CBM**

#1 "膀胱肿瘤"[不加权:扩展]

#2 "饮酒"[不加权:扩展]

#3 "饮酒"[常用字段:智能] OR "喝酒"[常用字段:智能] OR "酒精"[常用字段:智能] OR "酒"[常用字段:智能]

#4 "膀胱肿瘤"[常用字段:智能] OR "膀胱癌"[常用字段:智能] OR "膀胱肿物"[常用字段:智能]

#5 #4 OR #1

#6 #3 OR #2

#7 #6 AND #5

**CNKI**

#1 “膀胱肿瘤”[主题] OR “膀胱肿物”[主题] OR “膀胱癌”[主题]

#2 “饮酒”[主题] OR “喝酒”[主题] OR “酒”[主题] OR “酒精”[主题]

#3 #1 AND #2

**Supplementary Table 1** Studies excluded with reasons

| Number | Title | Reasons |
| --- | --- | --- |
| 1 | Cancer of the bladder and cigarette smoking, coffee and alcohol drinking in Lebanon. | Full-text could not be obtained |
| 2 | Alcoholism and cancer risk: a population-based cohort study. | Inappropriate alcohol categories |
| 3 | Adjustment disorder and type-specific cancer incidence: a Danish cohort study. | Inappropriate alcohol categories |
| 4 | Circulating concentrations of B group vitamins and urothelial cell carcinoma. | No appropriate associated data |
| 5 | Elevated cancer mortality in a German cohort of bitumen workers: extended follow-up through 2004. | Inappropriate outcome |
| 6 | The association of active smoking with multiple cancers: national census-cancer registry cohorts with quantitative bias analysis. | No appropriate associated data |
| 7 | Increased risk for urological cancer associated with anxiety disorder: a retrospective cohort study. | No appropriate associated data |
| 8 | Cancer risk in patients aged 30 years and above with type 2 diabetes receiving antidiabetic monotherapy: A cohort study using metformin as the comparator. | No appropriate associated data |
| 9 | Evaluation of a bladder cancer cluster in a population of criminal investigators with the Bureau of Alcohol, Tobacco, Firearms and Explosives-part 1: the cancer incidence. | No appropriate associated data |
| 10 | Evaluation of a bladder cancer cluster in a population of criminal investigators with the Bureau of Alcohol, Tobacco, Firearms and Explosives--part 2: the association of cancer risk and fire scene investigation. | No appropriate associated data |
| 11 | Nutritional quality of food as represented by the FSAm-NPS nutrient profiling system underlying the Nutri-Score label and cancer risk in Europe: Results from the EPIC prospective cohort study. | No appropriate associated data |
| 12 | Prospective associations between Vitamin D status, Vitamin D-related gene polymorphisms, and risk of tobacco-related cancers. | No appropriate associated data |
| 13 | Dietary intake of nutrients involved in one-carbon metabolism and risk of urothelial cell carcinoma: A prospective cohort study. | No appropriate associated data |
| 14 | Tobacco and the risk of cancer. Importance of kinds of tobacco. | Full-text could not be obtained |
| 15 | Mortality and incidence of cancer in a cohort of Swedish chimney sweeps: an extended follow up study. | No appropriate associated data |
| 16 | Cigarette smoking, educational level and total and site-specific cancer: a cohort study in men in Lithuania. | No appropriate associated data |
| 17 | Associations between diet and cancer, ischemic heart disease, and all-cause mortality in non-Hispanic white California Seventh-day Adventists. | No appropriate associated data |
| 18 | Quantitative exposure to metalworking fluids and bladder cancer incidence in a cohort of autoworkers. | Inappropriate alcohol categories |
| 19 | Effects of radiation and lifestyle factors on risks of urothelial carcinoma in the Life Span Study of atomic bomb survivors. | Inappropriate outcome |
| 20 | A common variant of the methylenetetrahydrofolate reductase gene (1p36) is associated with an increased risk of cancer. | No appropriate associated data |
| 21 | Associations between cancer incidence and alcohol/cigarette consumption among five ethnic groups in Hawaii. | No appropriate associated data |
| 22 | A large scale cohort study on cancer risks by diet--with special reference to the risk reducing effects of green-yellow vegetable consumption. | Full-text could not be obtained |
| 23 | Mortality in Japanese with life-styles similar to Seventh-Day Adventists: strategy for risk reduction by life-style modification. | Full-text could not be obtained |
| 24 | Cancer incidence in people with affective disorder: Nationwide cohort study in Taiwan, 1997-2010. | No appropriate associated data |
| 25 | Coffee drinking, mortality, and cancer incidence: results from a Norwegian prospective study. | No appropriate associated data |
| 26 | Trajectory of smoking and early bladder cancer risk among Korean young adult men. | No appropriate associated data |
| 27 | Cancer risk in hospitalised psoriasis patients: a follow-up study in Sweden. | No appropriate associated data |
| 28 | Total Fluid Intake and the Risk of Recurrence in Patients With Non-Muscle Invasive Bladder Cancer: A Prospective Cohort Study. | Inappropriate outcome |
| 29 | Cancer incidence among a large cohort of female Danish registered nurses. | No appropriate associated data |
| 30 | Relationship of serum uric acid to cancer occurrence in a prospective male cohort. | No appropriate associated data |
| 31 | Smoking cessation and survival in lung, upper aero-digestive tract and bladder cancer: Cohort study. | No appropriate associated data |
| 32 | The future burden of kidney and bladder cancers preventable by behavior modification in Australia: A pooled cohort study. | No appropriate associated data |
| 33 | Smoking, alcohol consumption, and cancer: A mendelian randomisation study in UK Biobank and international genetic consortia participants. | No appropriate associated data |
| 34 | A Prospective Investigation of Coffee Drinking and Bladder Cancer Incidence in the United States. | No appropriate associated data |
| 35 | Female mortality trends in Spain due to tumors associated with tobacco smoking. | No appropriate associated data |
| 36 | γ-Glutamyltransferase and cancer risk: The Korean cancer prevention study. | No appropriate associated data |
| 37 | Influence of diabetes on the risk of urothelial cancer according to body mass index: A 10-year nationwide population-based observational study. | Inappropriate outcome |
| 38 | A nested case-control study on alcohol drinking, tobacco smoking, and cancer. | Full-text could not be obtained |
| 39 | A prospective study of green tea consumption and cancer incidence, Hiroshima and Nagasaki (Japan). | No appropriate associated data |
| 40 | Cancer history and risk factors in healthy older people enrolling in the ASPREE clinical trial. | No appropriate associated data |
| 41 | Healthy Lifestyle Pattern is Protective Against 30-Yr Cancer Incidence in Men and Women: A Cohort Study. | No appropriate associated data |
| 42 | Blood lipid genetic scores, the HMGCR gene and cancer risk: A Mendelian randomization study. | No appropriate associated data |
| 43 | Air pollution from traffic and cancer incidence: A Danish cohort study. | No appropriate associated data |
| 44 | Diabetes mellitus and cancer incidence: the Atherosclerosis Risk in Communities (ARIC) cohort study. | No appropriate associated data |
| 45 | Aggregate Level Time Series Association between Alcohol Consumption and Cancer Mortality Rate. | Full-text could not be obtained |
| 46 | Is concordance with World Cancer Research Fund/American Institute for Cancer Research guidelines for cancer prevention related to subsequent risk of cancer? Results from the EPIC study. | No appropriate associated data |
| 47 | Fluid intake and the risk of urothelial cell carcinomas in the European Prospective Investigation into Cancer and Nutrition (EPIC). | Data from the same cohort |
| 48 | Obesity and cancer risk among white and black United States veterans. | No appropriate associated data |
| 49 | Association between rice consumption and risk of cancer incidence in the California Teachers Study. | No appropriate associated data |
| 50 | Second cancer following cancers of the buccal cavity and pharynx in Denmark, 1943-80. | Full-text could not be obtained |
| 51 | Adherence to the Mediterranean Diet and Risks of Prostate and Bladder Cancer in the Netherlands Cohort Study. | No appropriate associated data |
| 52 | Risk assessment of a cohort exposed to aromatic amines. Initial results. | Full-text could not be obtained |
| 53 | Risk factors for bladder cancer in a cohort exposed to aromatic amines. | No appropriate associated data |
| 54 | Cohort mortality study of 57,000 painters and other union members: a 15 year update. | No appropriate associated data |
| 55 | A cohort incidence study of workers exposed to perfluorooctanoic acid (PFOA). | No appropriate associated data |
| 56 | Increased lung and bladder cancer incidence in adults after in utero and early-life arsenic exposure. | Inappropriate alcohol categories |
| 57 | Cause-specific mortality and cancer incidence among 28,300 Royal Norwegian Navy servicemen followed for more than 50 years. | No appropriate associated data |
| 58 | The association between coffee consumption and bladder cancer incidence in a pooled analysis of the Miyagi Cohort Study and Ohsaki Cohort Study. | No appropriate associated data |
| 59 | Cancer incidence among patients with alcohol use disorders--long-term follow-up. | Inappropriate alcohol categories |
| 60 | Soy isoflavone intake and bladder cancer risk in Japan: From the takayama study. | No appropriate associated data |
| 61 | Prognostic significance of selected lifestyle factors in urinary bladder cancer. | Inappropriate outcome |
| 62 | Vitamin E and C supplementation and risk of cancer in men: posttrial follow-up in the Physicians' Health Study II randomized trial. | No appropriate associated data |
| 63 | Smoking history and cancer patient survival: a hospital cancer registry study. | Full-text could not be obtained |
| 64 | Cigarette smoking and cancer incidence risk in adult men: National Health Insurance Corporation Study. | No appropriate associated data |
| 65 | Flavonoid and lignan intake in relation to bladder cancer risk in the European Prospective Investigation into Cancer and Nutrition (EPIC) study. | No appropriate associated data |
| 66 | Are coffee, tea, and total fluid consumption associated with bladder cancer risk? Results from the Netherlands Cohort Study. | No appropriate associated data |
| 67 | Nitrate intake does not influence bladder cancer risk: the Netherlands cohort study. | No appropriate associated data |
| 68 | Risk assessment of secondary primary malignancies in nasopharyngeal carcinoma: A big-data intelligence platform-based analysis of 6,377 long-term survivors from an endemic area treated with intensity-modulated radiation therapy during 2003-2013. | No appropriate associated data |
| 69 | Fluid intake and risk of bladder cancer in the Nurses' Health Studies. | No appropriate associated data |
| 70 | 上海制革业联苯胺染料接触者中癌症发病的队列研究. | No appropriate associated data |
| 71 | 肥胖相关身体测量指标与恶性肿瘤发病风险的前瞻性队列研究. | No appropriate associated data |
| 72 | BMI与恶性肿瘤发病风险的前瞻性队列研究. | No appropriate associated data |
| 73 | 麻醉方式及其相关因素对膀胱癌膀胱部分切除术患者年内复发影响的研究. | Inappropriate outcome |
| 74 | 不同麻醉方式及其他因素对膀胱癌切除术患者术后复发的影响. | Inappropriate outcome |
| 75 | Cancer incidence among patients with alcohol use disorders - Long-term follow-up. | No appropriate associated data |

**Supplementary Table 2** Quality assessment of included studies (Newcastle Ottawa Scale)

| **Study** | **Selection** | | | | **Comparability** | **Outcome** | | | **Quality scores** |
| --- | --- | --- | --- | --- | --- | --- | --- | --- | --- |
|  | Representativeness of the exposed cohort | Selection of the non-exposed cohort | Ascertainment of exposure | Demonstration that outcome of interest was not present at start of study | Comparability of cohorts on the basis of the design or analysis^&^ | Assessment of outcome | Was follow-up long enough for outcomes to occur^#^ | Adequacy of follow up of cohorts^¶^ |  |
| Mills 1991 | * | * | * | * | ** | * | * | * | 9 |
| Chyou 1993 | * | * | * | * | * | * | * | * | 8 |
| Michaud 1999 | - | * | * | * | ** | * | * | * | 8 |
| Zeegers 2001 | * | * | * | * | * | * | * | * | 8 |
| Djoussé 2004 | * | * | * | * | ** | * | * | * | 9 |
| Allen 2009 | * | * | * | * | ** | * | * | * | 9 |
| Botteri 2017 | * | * | * | * | ** | * | * | * | 9 |
| Masaoka 2017 | * | * | * | * | ** | * | * | * | 9 |
| Park 2017 | - | * | * | * | ** | * | - | * | 7 |

&We selected “age, sex, and smoking” as the most important adjusting factors.

#A mean duration of follow-up of at least 3 years was considered as long enough for the outcome to occur in this meta-analysis.

¶It was regarded adequate when the follow-up rate was at least 90%.

**Supplementary Table 3** Summary of the risk of bladder cancer with different alcohol consumption (light, moderate, and heavy) in different subgroups

| Subgroup | Alcohol exposure | Cases | HR | 95%CI | Adjusted confounders |
| --- | --- | --- | --- | --- | --- |
| Male | None | 84 | 1 | -- | Age, sex, smoking status, energy intake, body mass index, physical activity and educational level completed |
|  | Light | 497 | 1.13 | 0.94-1.35 |  |
|  | Moderate | 276 | 1.24 | 0.96-1.59 |  |
|  | Heavy | 416 | **1.23** | **1.02-1.48** |  |
| Female | None | 107 | 1 | **--** |  |
|  | Light | 289 | **0.79** | **0.66-0.96** |  |
|  | Moderate | 76 | 0.85 | 0.62-1.16 |  |
|  | Heavy | 57 | 0.94 | 0.67-1.3 |  |
| Beer | None | 530 | 1 | -- | Age, sex, study center, smoking status, energy intake, body mass index, physical activity and educational level completed |
|  | Light | 1051 | 0.97 | 0.87-1.08 |  |
|  | Moderate | 103 | 0.96 | 0.76-1.21 |  |
|  | Heavy | 118 | 1.13 | 0.92-1.41 |  |
| Beer-male | None | 271 | 1 | -- |  |
|  | Light | 788 | 0.97 | 0.84-1.11 |  |
|  | Moderate | 101 | 1 | 0.78-1.29 |  |
|  | Heavy | 113 | 1.12 | 0.89-1.42 |  |
| Beer-female | None | 259 | 1 | -- |  |
|  | Light | 263 | 0.99 | 0.82-1.18 |  |
|  | Moderate | 2 | 0.29 | 0.07-1.21 |  |
|  | Heavy | 5 | 1.7 | 0.69-4.18 |  |
| Wine | None | 432 | 1 | -- |  |
|  | Light | 1022 | 0.97 | 0.87-1.09 |  |
|  | Moderate | 119 | 0.92 | 0.73-1.16 |  |
|  | Heavy | 229 | 1.05 | 0.88-1.25 |  |
| Wine-male | None | 277 | 1 | -- |  |
|  | Light | 711 | 1.01 | 0.88-1.16 |  |
|  | Moderate | 84 | 0.86 | 0.66-1.13 |  |
|  | Heavy | 201 | 1.06 | 0.87-1.29 |  |
| Wine-female | None | 155 | 1 | -- |  |
|  | Light | 311 | 0.91 | 0.76-1.1 |  |
|  | Moderate | 35 | 1.1 | 0.73-1.65 |  |
|  | Heavy | 28 | 0.96 | 0.63-1.47 |  |
| Liquor or spirits | None | 539 | 1 | -- |  |
|  | Light | 1124 | 1.05 | 0.94-1.17 |  |
|  | Moderate | 88 | 1.17 | 0.91-1.5 |  |
|  | Heavy | 51 | 1.26 | 0.93-1.72 |  |
| Liquor or spirits-male | None | 318 | 1 | -- |  |
|  | Light | 833 | 1.09 | 0.95-1.25 |  |
|  | Moderate | 78 | 1.24 | 0.94-1.62 |  |
|  | Heavy | 44 | 1.3 | 0.93-1.83 |  |
| Liquor or spirits-female | None | 221 | 1 | -- |  |
|  | Light | 291 | 0.96 | 0.8-1.16 |  |
|  | Moderate | 10 | 0.88 | 0.46-1.7 |  |
|  | Heavy | 7 | 1.15 | 0.52-2.5 |  |
| Never smokers | None | 57 | 1 | -- | Age, sex, smoking status, energy intake, body mass index, physical activity and educational level completed |
|  | Light | 200 | 1 | 0.77-1.28 |  |
|  | Moderate | 60 | 1.14 | 0.76-1.7 |  |
|  | Heavy | 41 | 0.86 | 0.56-1.32 |  |
| Smokers | None | 134 | 1 | -- |  |
|  | Light | 578 | 0.93 | 0.8-1.08 |  |
|  | Moderate | 289 | 1.03 | 0.82-1.29 |  |
|  | Heavy | 430 | 1.1 | 0.93-1.3 |  |


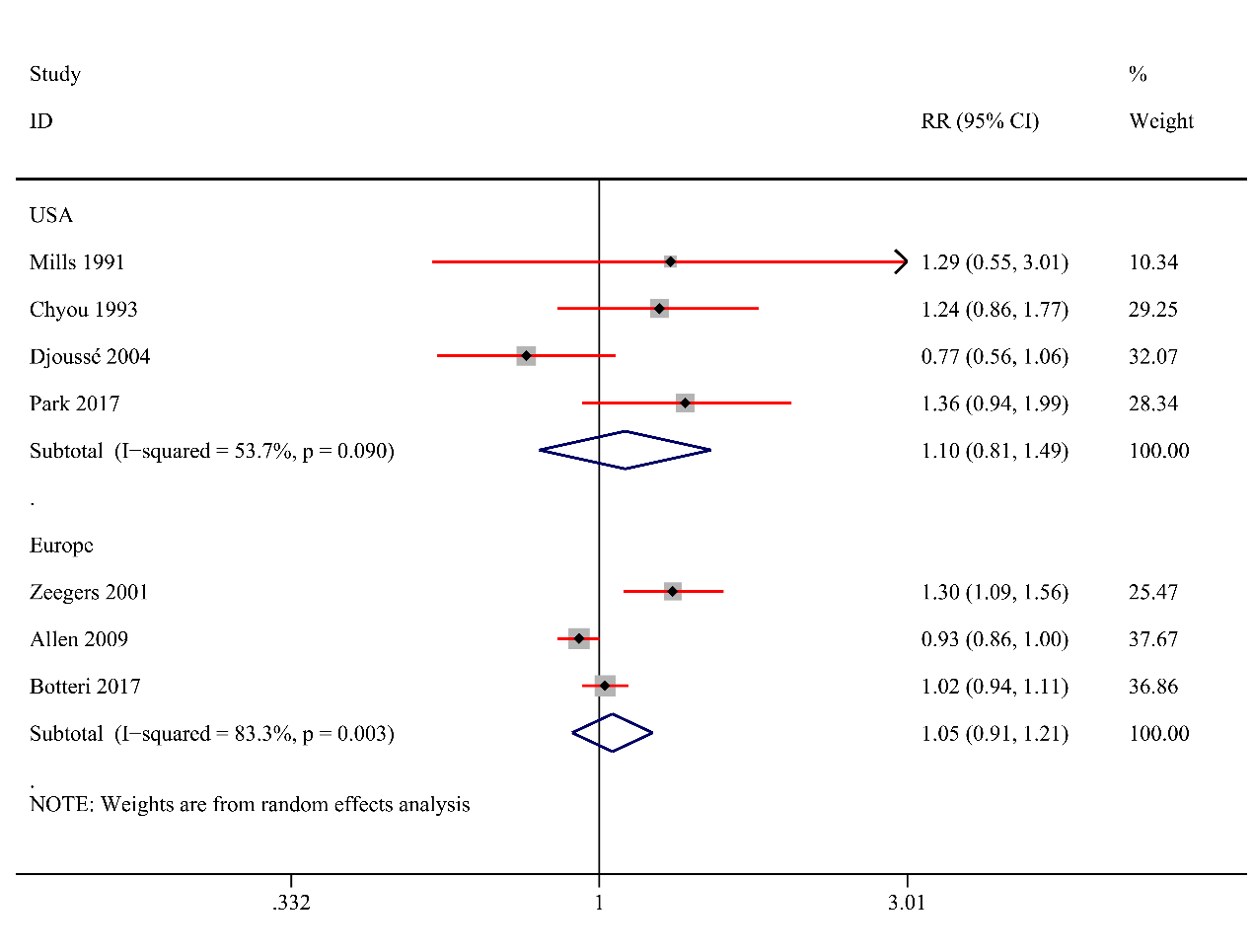


**Supplementary Figure 1** Forest plot of relative risk (RR) of bladder cancer for alcohol consumption (Any versus none) in different regions

**
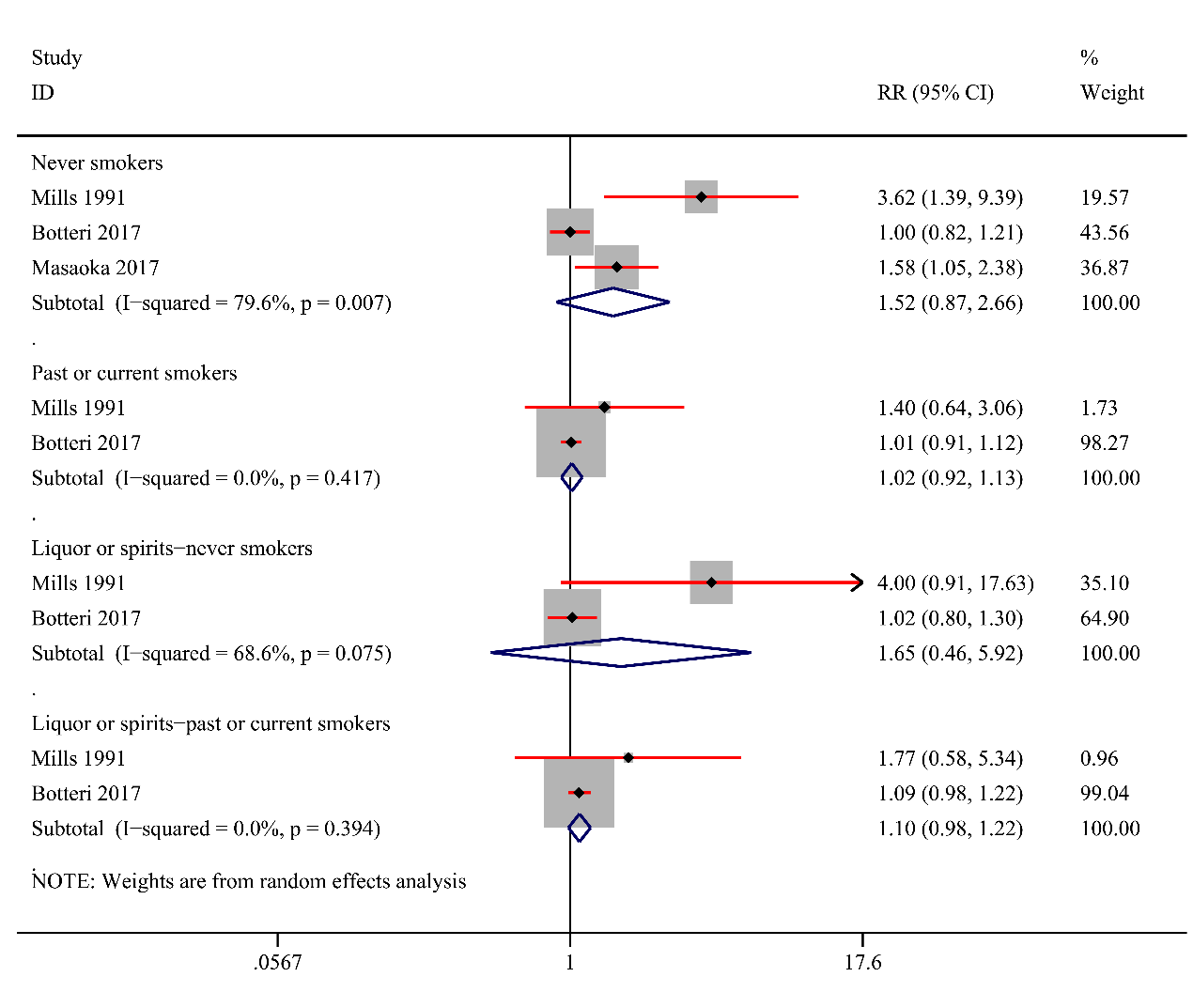
**

**Supplementary Figure 2** Forest plot of relative risk (RR) of bladder cancer for alcohol consumption (Any versus none) in population with different smoking status

**
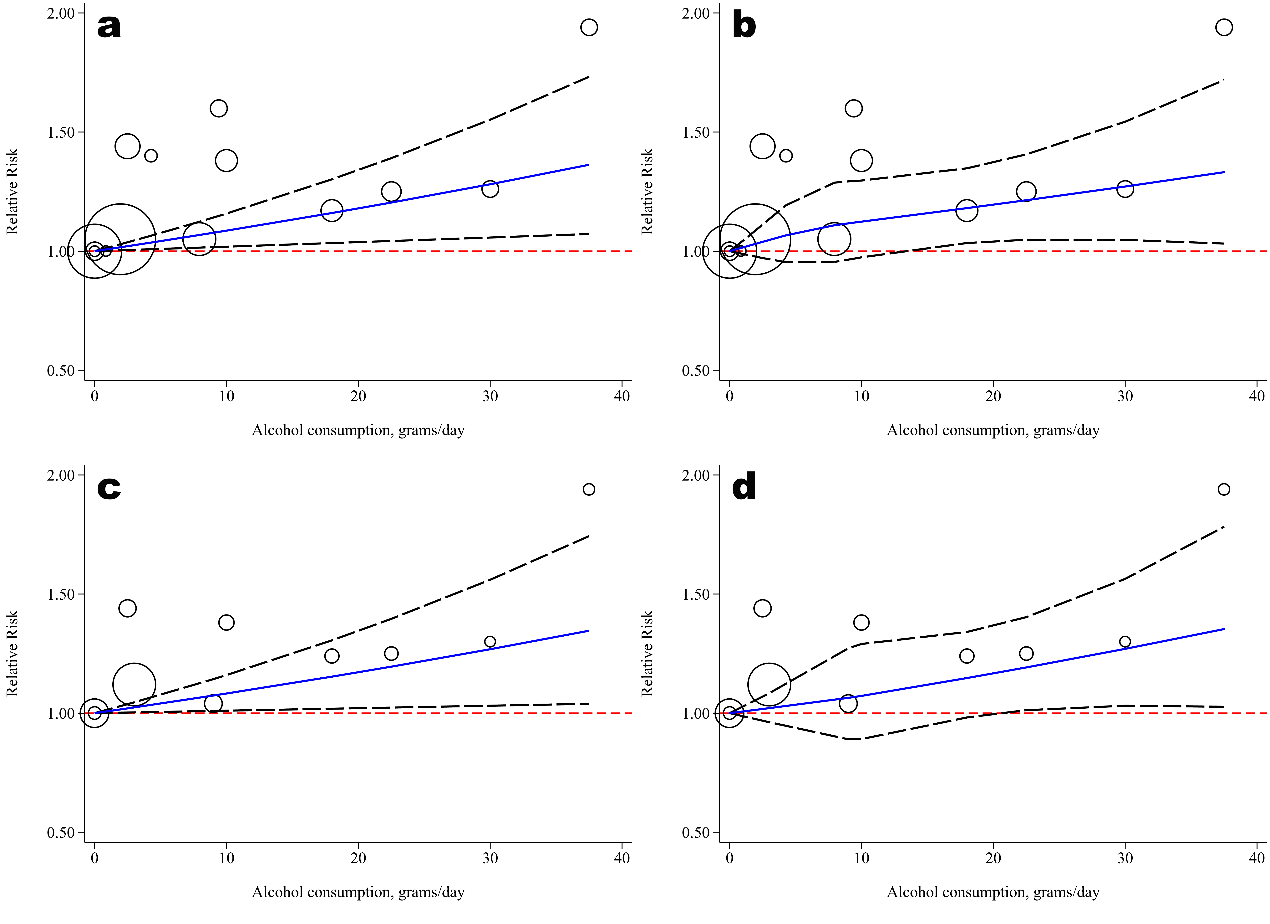
**

**Supplementary Figure 3** Sensitivity analysis of linear and nonlinear fitting of alcohol consumption and risk of bladder cancer (a: linear association in entire population who consumed alcohol for liquor or spirits; b: nonlinear association in entire population who consumed alcohol from liquor or spirits; c: linear association in males who consumed alcohol form liquor or spirits; d: nonlinear association in males who consumed alcohol from liquor or spirits) (Bubbles were weighted by the number of cases at each dose point)
